# The Use of “Literary Fiction” to Promote Mentalizing Ability

**DOI:** 10.1371/journal.pone.0160254

**Published:** 2016-08-04

**Authors:** Maria Chiara Pino, Monica Mazza

**Affiliations:** 1 Department of Life, Health and Environmental Sciences, University of L’Aquila, L’Aquila, Italy; 2 Department of Applied Clinical Sciences and Biotechnology, University of L’Aquila, L’Aquila, Italy; Oregon Health and Science University, UNITED STATES

## Abstract

Empathy is a multidimensional process that incorporates both mentalizing and emotional sharing dimensions. Empathic competencies are important for creating interpersonal relationships with other people and developing adequate social behaviour. The lack of these social components also leads to isolation and exclusion in healthy populations. However, few studies have investigated how to improve these social skills. In a recent study, Kidd and Castano (2013) found that reading literary fiction increases mentalizing ability and may change how people think about other people’s emotions and mental states. The aim of our study was to evaluate the effects of reading literary fiction, compared to nonfiction and science fiction, on empathic abilities. Compared to previous studies, we used a larger variety of empathy measures and utilized a pre and post-test design. In all, 214 healthy participants were randomly assigned to read a book representative of one of three literary genres (literary fiction, nonfiction, science fiction). Participants were assessed before and after the reading phase using mentalizing and emotional sharing tests, according to Zaki and Ochsner’ s (2012) model. Comparisons of sociodemographic, mentalizing, and emotional sharing variables across conditions were conducted using ANOVA. Our results showed that after the reading phase, the literary fiction group showed improvement in mentalizing abilities, but there was no discernible effect on emotional sharing abilities. Our study showed that the reading processes can promote mentalizing abilities. These results may set important goals for future low-cost rehabilitation protocols for several disorders in which the mentalizing deficit is considered central to the disease, such as Autism Spectrum Disorders and Schizophrenia.

## Introduction

Empathy is the capacity to share the physiological and emotional states of other people [1, 2, 3]. Empathy has been recently described as including two dimensions: mentalizing and emotional sharing [4–7]. Mentalizing is the ability to understand what others are thinking or feeling, while the emotional sharing dimension includes the capacity to emotionally resonate with other people’s feelings [8]. Empathy is a necessary capacity to guide interpersonal relationships and it enables us to interact successfully in social groups [9]. Thus, people with this competence is more able to adapt and it prevents them from suffering, isolation, loneliness and exclusion. For this reason, it is important to design an intervention aimed at improving empathic ability in healthy populations.

Recent studies [10, 11] have pointed towards reading literary texts as a means of enhancing empathic abilities. The abilities to read, understand and enjoy stories imply both cognitive (e.g., attention and memory) and affective processes (e.g., emotion and empathy) [12]. Kumschick et al. [13] showed that reading and discussing children’s books with emotional content increases children’s emotional competences. Their findings underscore the role that reading can play in supporting children’s development of emotional and social skills [13, 14]. Moreover, reading fiction also seems to improve mentalizing among adults[15, 16]. Kidd and Castano [17] demonstrated that reading literary fiction leads to better performance on assessments of mentalizing compared to reading nonfiction, popular fiction, or nothing at all. Their study [17] consisted of five experiments in which the authors evaluated the effects of reading literary fiction compared to reading nonfiction (Experiment 1) and popular fiction (Experiments 2–5) on mentalizing skills using three different measuresThis study [17] showed that only reading literary fiction affected mentalizing. Recently, a study has extended Kidd and Castano’ s results [16]. The authors [16] evaluated the effects of reading on both social and non-social cognition. In this study [16] the participants read two literary short stories and two nonfiction articles: one of the texts used in each condition was taken directly from Kidd and Castano’ s materials [17]. Moreover, the authors [16] used only two tests: the *Reading the Mind in the Eyes Task* as a mentalizing measure [18] and the *Intuitive Physics Test* [19] as a non-social measure. This study revealed the specificity of the effect to social cognition, and it controlled for pre-reading mentalizing performance. However, the existing studies remain limited by a restricted number of measures of mentalizing and empathy.

The present research builds on these prior studies by including a more comprehensive battery of mentalizing and empathy tests (all those available in Italy and already used in studies of healthy and clinical Italian populations) during the pre and post reading phases. The pre reading phase offers guarantees with regard to the equivalence of the three groups of participants prior to the manipulation, it is nonetheless useful to further demonstrate that the three groups do not differ a-priori on important measures of empathy. To avoid a learning effect between the pre- and post- measures, we used different empathic measures in the two phases (see Table 1). Specifically, regarding mentalizing, we used measures that evaluated three aspects of this competence: cognitive empathy, perspective taking and mind-reading according to Zaki and Ochsner’ s model [8]. Regarding emotional sharing components, we assessed: affective empathy, shared self-other representations, and emotional contagion. Given the complexity of empathic abilities, a more extensive, multi-faceted investigation of how they are affected by reading fiction is important. In particular, a more nuanced understanding of the effects of reading is necessary to inform potential rehabilitation treatments for disorders in which a deficit of empathy is central, such as Autism Spectrum Disorders (ASDs) and schizophrenia.

**Table 1:** Pre and post reading phases measures.

*Mentalizing*		*Emotional Sharing*	
*PRE-READING*	*POST-READING*	*PRE-READING*	*POST-READING*
Cognitive Sub-scale (BES)	Cognitive Empathy (MET)	Affective Sub-scale	Explicit Emotional Empathy (MET)
Fantasy items (IRI)	Emotion Attribution Task	Personal Distress items (IRI)	Implicit Emotional Empathy (MET)
Perspective Taking items (IRI)	Cognitive Empathy (EQ)	Empathic Concern items (IRI)	Emotional Empathy (EQ)
Attribution of intention task	Social Skills (EQ)		
Eyes Task	Faces test		
Advanced Theory of Mind	First and second order false belief test		

*Legend*: BES = Basic Empathy Scale; MET = Multifaceted Empathy Test; IRI = Interpersonal Reactivity Index; EQ = Empathy Quotient

In the present study, we focused on three comparisons: (1) literary fiction versus nonfiction; (2) literary fiction versus science fiction; and (3) nonfiction versus science fiction. The literary fiction versus nonfiction condition was used for two reasons.

This condition is only briefly reported in Kidd and Castano’ s study [17], and perhaps nonfiction books (at least those that we have selected for our study) could have an effect on the emotional sharing component because we have chosen the nonfiction books that narrate true stories of illness, death or pain. It is also important to underline that there are also nonfiction books such as essays about dinosaurs or geology in which may to lack a social focus.

The comparison between literary fiction and science fiction was performed because even if both are fiction, these two genres may require different types of imagination and creativity. Indeed, most science fiction books narrate stories based on abstract topics such as space, time, the destiny of man, our place in the universe etc. On the other hand, in literary fiction, the readers can probably imagine the feelings and thoughts of characters, but the context and characters are inspired by real situations. For this reason, we think that is easier for readers of literary fiction identify oneself with the characters of the story, because, literary fiction is more realistic compared to science fiction. In addition, while in most literary fiction the emotional experiences of realistic characters and the relationship between the people are in the foreground compared to events narrate, science fiction may instead focus attention on differences between the real world and the imaginary world and we believe that the emotional aspects of the characters of the science fiction books are in the background compared to the plot of the story. Therefore, reading literary fiction would most likely lead readers to focus on the understanding of the mental and emotional states of the characters. Finally, we compared literary nonfiction with science fiction because these are completely opposite genres. The former narrates true stories, while the latter is based on fantasy and may involve different cognitive mechanisms. Compared to other studies [16, 17], we added the science fiction condition in order to use a category of fiction which is unrealistic and the scenes are often foreign to one's own experiences. Hence the reader is forced to imagine them. Literary fiction and nonfiction may both require more emotional engagement compared to science fiction books. Moreover, we hypothesize that this lack of emotional engagement in science fiction books could make them more suitable to potential treatments involving individuals with autism [20, 21].

## Method

### Subject selection and data collection

The study was carried out using 214 participants (undergraduate students attending the Master Degree course in Psychology at the University of L’Aquila). The participants were randomly assigned to read one of six short books (two literary fiction, two nonfiction and two science fiction; see below for details) with pre-and post-reading phase assessments. The University of L’Aquila independently conducted the randomisation procedures. A random-number table was used to generate lots that were drawn in sealed envelopes, each assigning the respective participants to the literary fiction, nonfiction or science fiction groups. The random assignment ensures that there are not systematic differences between the groups on a specific measure, which makes it more likely that the groups will be equivalent prior to some manipulation. The students were asked to participate in the intervention as part of their course programme requirements for psychometrics, and participation in this experiment allowed them to obtain an academic credit. In the pre- and post-reading phases, the participants underwent an empathic abilities assessment through a collective administration of tests. The collective administration was characterised by minimising interactions between the examiner and the subject, as well as the need to plan the sequence in which the tests were to be administered simultaneously.

All empathy measures were implemented using the Powerpoint programme, and for each test a psychologist (the first author of this paper) and the professor of the psychometrics course (the second author) gave the instructions.

As part of the response protocol, the participants had to provide sociodemographic characteristics (age and gender) and a pseudonym (e.g., a cartoon name) to ensure anonymity. We checked that the participants had not already read the book assigned. In addition, we selected books of the same reading level, difficulty and length. The language of all the books was Italian, and all the participants were native Italian speakers. The participants read the book in their own home over the course of a week.

After the reading phase, each participant completed a questionnaire with three questions to evaluate how effective the participant was in reading the assigned book. For inclusion criteria, the participants had to answer to all three questions on the questionnaire correctly. If the participants only responded correctly to one or two questions, he or she was excluded from the study. In fact, initially, the total sample consisted of 250 participants. Thirty-six students were excluded because they did not respond to all three questions, and it was unclear whether or not they had read the book.

The time between the pre- and post-reading phases was 14 days. The participants had one week to read the book. After the pre- reading phase, the participants had from 1 to 5 days to buy the book; only when all the participants had the assigned book could the reading of it begin.

Therefore, to avoid a learning effect we decided to use different empathic measures during the pre and post-reading phases (see Table 1).

The study was conducted at the University of L'Aquila, according to the principles established by the Declaration of Helsinki.

Ethical approval was obtained by the University of L'Aquila. Informed consent was obtained from all participants before the investigation in written form.

## Materials

### Reading stimuli

The criteria used for the selection of books were:

1. number of pages (the books have about same number of pages)
2. simplicity of language
3. availability in Italian libraries
4. awards
5. price
6. most read according to online surveys among young readers

### Literary fiction books

- “*Tenth of December”* (*Dieci dicembre—*Italian title)[22]: 10 narrative stories that take place in suburban areas or small towns. In the book there are stories of normal families whose everyday life is changed, for instance by the return of a son or a war. The characters of each story must choose among egoism, compassion, self-esteem or sacrifice. The book has 222 pages. It was voted the best book of 2013 according to the following classifications: votes from readers (NY Times bestseller and Amazon, the votes of the users of the social networking site Goodreads) and critics (Guardian, Washington Post, Time).
- *“Io e Te”*(only Italian version) [23]: the protagonist of the story is Lorenzo, an adolescent who is very introverted and has trouble socializing with his peers. Lorenzo was not invited by his friends to spend a week in the mountains, but he tells a lie to his mother and says that he has to go to the mountains with his friends. Really, he hides in the cellar, living a week alone, free from all social difficulties and the harassment of his companions. During this week he meets his half-sister, his father's daughter and discovers new aspects of his personality. There are 116 pages in total. This book has received several awards as has the film version -such as “David di Donatello”, “Nastro D’Argento dell’anno”-the Italian awards.

### Nonfiction books

- *“Intervista ad Oriana Fallaci”* (only Italian version)[24]: biography of a woman who had the courage to write the truth about herself, from the passion for her work (i.e., journalism) to illness (i.e., cancer). The book is 126 total in length. In addition, we selected this book because the author is one of the most well-known Italian journalists and in the book Oriana talks about her personal and private life. The book had much success in Italy, selling 800,000 copies in just one summer.
- “*Wave”* (*Onda—*Italian title) [25]: the book recounts the life and memories of the author after a tsunami. It is written in the first person narrative style. There are 204 total pages. The author won the PEN/Ackerley Prize in 2014 for this book. This book has received several awards: one of The New York Times's 10 Best Books of the Year, a Christian Science Monitor Best Nonfiction Book, a Newsday Top 10 Books’ pick, a People magazine Top 10 pick, a Good Reads Best Book of the Year, and a Kirkus Best Nonfiction Book.

### Science fiction books

- *Troika* (*L’ultimo Cosmonauta-* Italian title) [26]: a narrative story of a trio of Russian men and women confronting an enigma named "Matryoshka", a vast alien construct whose periodic appearances generate terror, wonder, and endless debate. There are 112 total pages. Troika was shortlisted for the 2011 Hugo Award for best novella.
- *Fahrenheit 451 (In the Italian Version the title is the same)* [27]: presents a future American society where books are outlawed. The main character is Guy Montag, a "fireman" hired to burn the possessions of those who read outlawed books. When his wife, Mildred, attempts suicide and Clarisse, his eccentric young neighbour, suddenly disappears, Montag begins to question everything he has ever known. He starts hiding books in his home, and when his pilfering is discovered, the fireman has to run for his life. There are 210 total pages. This book is the winner of many awards, including the Grand Master Award for Science Fiction.

## Empathic Measures

### Mentalizing tasks

For the pre-reading phase, we used the following measures:

*Basic Empathy Scale- Cognitive Empathy Sub-scale*(BES- CES) [28, 29]: the BES- CS is comprised of nine items (N° 3, 6, 9, 10, 12, 14, 16, 19, 20) and measures understanding of another person’s emotion [28]. Examples of items on the CES are: “I can understand my friend’s happiness when she/he does well at something” and “When someone is feeling down I can usually understand how they feel”. The participants had to give their ratings on a five-point Likert type scale (1 = Strongly Disagree, 2 = Disagree, 3 = Neither Agree nor Disagree, 4 = Agree, 5 = Strongly Agree). The BES has demonstrated good validity [28, 29]. The Cronbach’s alpha coefficient was calculated to examine the internal consistency of both sub-scales (α = .74 for CES). According to Jolliffe and Farrington’ study [28], for the cognitive scale, males had a mean of 32.2 (standard deviation = 5.1), and females had a mean of 35.0 (standard deviation = 3.9).

*The Eyes Task* is a revised version of the “Reading the Mind in the Eyes Test” [18]. Baron-Cohen [18] considers this test as a ‘pure’ theory of mind test, at an advanced level. In brief, participants are given 36 photographs depicting the ocular area in an equal number of different actors and actresses. At each corner of every photo, four emotional descriptors (e.g., dispirited, bored, playful or comforting), are printed, only one of which (the target word) correctly identifies the depicted person's mental state, while the others are included as foils. The test is scored by totalling the number of items (photographs) the participant correctly identifies. Therefore, the maximum total score is 36. The mean and standard deviation for the student sample is, respectively, 28.0 and 3.5 (for details see [18]).

*Interpersonal Reactivity Index cognitive sub-scales* (IRI): the IRI, which Davis [30] developed, is the most frequently used self-administered instrument to assess the different components of empathy. The IRI cognitive sub-scales include 14 items divided into two scales: fantasy (FS) and perspective taking (PT). The FS sub-scale evaluates the tendency of the individual to identify him or herself with fictitious personages, such characters from books, films, or video games (e.g., “When I am reading an interesting story or novel, I imagine how I would feel if the events in the story were happening to me”). The standardized alpha coefficients for the FS scale are .78 for males and .79 for females [30]. The PT sub-scale evaluates the tendency of an individual to spontaneously adopt the psychological point-of-view of another person (e.g., “I sometimes find it difficult to see things from the other guy's point of view”). The standardized alpha coefficients for PT are .71 for males and .75 for females [30].

Each answer can vary from 0 (*does not describe me well*) to 4 (*describes me very well*). The scores of each sub-scale are calculated individually. The IRI does not provide a total score because each sub-scale evaluates an independent component of empathy [3, 31, 32].

*Advanced Theory of Mind Task*. This task is an Italian adaptation of a cognitive task that Blair and Cipolotti [33] used and was proposed in the literature by Happè [34]. The Italian task consists of a short version of 13 vignettes, each accompanied by two questions: the comprehension question “*Was it true; what X said*?”, and the justification question “*Why did X say that?*”. The 12 story-types include Lie, White Lie, Joke, Pretence, Misunderstanding, Double Bluff, Contrary Emotions, Figure of speech, Appearance/Reality, Forgetting, Irony, Persuasion. Each subject earns a score ranging from 0 to 1 for each question. The maximum score is 13. Happè [33] used the term “advanced” to refer to a story that contains the comprehension question, where the key questions in the task either concerned a character’s mental states (the experimental condition). Happè’s task is more advanced than previous tests of theory of mind. The maximum score is 13 and the cut-off for the Italian normative sample is equivalent to 12.

*Attribution of Intentions Task* [35]: the concept of attribution of intentions to others is a central element of what is often referred to as theory of mind. Cartoon-type stimuli depicting sequences of intentional actions have thus been utilized to evaluate mentalizing performance. Each scenario showed a character performing a very simple action in order to facilitate identification of the intention that motivated it. This nonverbal test used a picture-sequencing task that includes three conditions: (1) attribution of intentions; (2) physical causality with human figures and (3) a physical causality condition involving objects only. In our experiment, we only consider the attribution of intentions condition (A and B series). In this condition, the stories involve human agents whose intentions must be inferred to select the picture missing from the sequence. The stories were designed to depict simple first-order intentional behaviour. Each subject can earn a score ranging from 0 to 1 for each story. The score range for this task is 0–28.

For the post-reading phase, we used the following measures *Empathy Quotient Scale* (EQ) is a self-report measure that Baron-Cohen et al. [36] developed to measure the different aspects of empathy [37–40]. The cognitive dimensions of empathy are evaluated by two sub-scales of the EQ: cognitive empathy (CE) and social skills (SS), which measure, respectively, the capacity to take the perspective of the other person and some regulatory mechanisms that keep track of the origins of self and other-feelings. The means (and standard deviations) for CE and SS are, respectively 8.9 (4.3) and 4.7 (2.3) with a Cronbach’s alpha of 0.85 and 0.55 [38].

*First and Second Order False Belief Test* [41]. These tests were designed to evaluate the ability to solve problems involving either (1) first-order attributions of false belief (of form “A” thinks “X”) and (2) second-order attributions of false belief (of form “A” thinks “B” thinks “X”). We think that the second-order false belief test is a form of *Advanced Theory of Mind*, because the questions of both tests concern the capacity of the people to understand the cause of the behaviour of the character in the story (“*Why did X say this*?”). An example of first-order theory of mind is “The washing machine task” [41,42]; whereas an example of second-order theory of mind is “The wallpaper” [41]. Each subject can earn a score ranging from 0 to 1 for each question.

*Multifaceted Empathy Test (MET)—Cognitive Empathy (CE)*: the CE component evaluates the capacity of the subjects to infer the emotional and mental states of the individuals shown in the image [2, 6, 43]. This test consists of a series of photographs that depict people in emotionally charged situations. In these pictures, taken from the International Affective Picture System (IAPS) [44], the stimuli show individuals feeling different emotions: positive emotions (25 pictures that include emotions such as happiness, positive surprise) and negative emotions (25 pictures that include emotions such as sadness, anger, disappointment). Positive and negative emotions are presented in random order. All the stimuli were displayed on a black screen. For each of the 50 stimuli presented, the subjects are required to infer the mental and emotional state of the individual/s depicted in the picture (*What kind of emotion/mental state is/are the person/people feeling?*). The score of cognitive empathy is the sum of the correct response (maximum score is 50) [6].

*Faces Test* [45]: an actress was invited to pose using various facial expressions and her face was photographed under controlled and standardized lighting conditions, with her head always facing forward. The test is comprised of 10 pictures that represent seven “basic” emotions (happy, surprise, sad, angry, afraid, disgust, and distress; surprise, happy, and angry were repeated, using new poses, to make a the set of 10 pictures), and 10 pictures that represent nine “complex” mental states (scheming, guilt, thoughtful, admiring, quizzical, flirting, bored, interested, and arrogant; interested was repeated in the set of complex mental states, to make a set of 10, although a new pose was used). Under each photo (full face, eyes, or mouth) a target word was typed, describing the mental state the actress was demonstrating. These words were chosen by a panel of four independent judges (two male, two female), and only those terms that produced unanimous agreement were used.

*Emotion Attribution Task* [33]: This is an assessment of the ability to represent the emotions of other people. In this task, the participant is presented with 58 short stories describing an emotional situation and is required to provide an emotion describing how the main character might feel in that situation. The sentences are designed to elicit attributions of positive and negative emotions. The task is scored according to the number of correct attributions. Validation studies are lacking for this test [33].

### Sharing tasks

For the pre-reading phase, we used the following measures: *Basic Empathy Scale-Affective Empathy Sub-scale* (AES) [28, 29]: the AES is comprised of 11 items (N° 1, 2, 4, 5, 7, 8, 11, 13, 15, 17, 18) that measure congruence with another person’s emotions [18]. Example of items on the AES are: “My friend’s emotions don’t affect me much” or “I often get swept up in my friend’s feelings”. The α coefficient for AES is 0.86 [28]. According to Jolliffe and Farrington’ s study [28], for the affective empathy scale, males had a mean of 32.1 (standard deviation = 6.5), while females had a mean of 40.3 (standard deviation = 5.8).

*Interpersonal Reactivity Index- Affective sub-scales* [30]: The IRI affective sub-scales include a total of 14 items divided into two scales the ‘‘personal discomfort” (PD) and the ‘‘empathic concern” (EC) sub-scales. The PD sub-scale evaluates self-oriented anxiety and discomfort resulting from tense personal situations (e.g., I tend to lose control during emergencies). The standardized alpha coefficients for PD are .77 for males and .75 for females. The EC sub-scale refers to feelings of compassion, tenderness and concern for other people (e.g., “When a friend tells me about his good fortune, I feel genuinely happy for him”). The standardized alpha coefficients for EC are .68 for males and .73 for females [29].

For the post-reading phase, we used the following measures *Empathy Quotient- Emotional sub-scale* [46, 38]. The EQ Emotional sub-scale, also called *Emotional Reactivity*, consists of item numbers 4, 8, 12, 14, and 35. An example of an item is “I find it hard to understand how to behave in a social situation”. The mean and standard deviation scores are for the general population are 10.3 and 3.2. The Cronbach’s alpha for the EQ-ER was 0.65 [38].

*Multifaceted Empathy Test (MET)*- Emotional Empathy (EE) components [2, 6, 43] is assessed by instructing subjects rate their level of empathic concern for the individuals displayed in the images using a nine-point Likert scale. The EE component is divided into implicit and explicit components. For each stimulus presented, the subjects are required to answer two questions: “*How aroused does this picture make you feel*?” for implicit EE and *“How strong is the emotion you feel about this person?”* for explicit EE. The score for implicit and explicit emotional empathy range between 1 and 9, where 1 and 9 correspond to the minimum and maximum levels of arousal (implicit empathy) and empathic concern (explicit empathy), respectively. The final score for the separate conditions (implicit and explicit) correspond to the mean of all responses [6].

## Results

An ANOVA was used to analyse sociodemographic, mentalizing and emotional sharing measures during the pre- and post-reading phases. The results on the sociodemographic and empathic measures during the pre-reading phase showed no significant differences among groups (see Table 2).

**Table 2:** Socio-demographic data and empathic measures results of the participants. Mean scores (and standard deviations) and results of the statistical analyses are shown separately for three groups shown. The results of the statistical analyses (ANOVA and post-hoc) are also shown.

	Literary Fiction (G1) N = 74	Nonfiction (G2) N = 67	Science Fiction (G3) N = 73	Group1 vs Group2 vs Group 3				Effect Size
	*Mean (SD)*	*Mean (SD)*	*Mean (SD)*	*F*	P	P	P	*ŋ*^*2*^_*p*_
					G1 vs G2	G1 vs G3	G2 vs G3	
**Age**	23.96 (7.46)	21.91 (2.91)	23.43 (5.58)	2.63	0.09	1	0.27	0.02
**Education (years)**	15.38 (0.68)	15.46 (0.78)	15.57 (1.53)	0.54	1	0.96	0.83	0.05
**Raven’s Matrices**	32.67 (2.37)	32.81 (3.19)	31.81 (5.11)	1.50	1	0.53	0.27	0.01
**Gender**	15 M; 59 F	18 M; 48 F	12 M; 61 F	-	-	-	-	-
***Mentalizing***								
**Cognitive Empathy (BES)**	30.34 (6.96)	29.54 (6.49)	29.76 (6.34)	F_2,211_ = 0.28	0.76	0.87	0.98	0.003
**Fantasy (IRI)**	12.15 (5.74)	11.76 (5.93)	11.06 (5.94)	F_2,211_ = 0.67	0.91	0.51	0.76	0.006
**Perspective Taking (IRI)**	9.64 (4.49)	10.43 (4.92)	10.09 (4.66)	F_2,211_ = 0.52	0.59	0.85	0.71	0.005
**Attribution Intention Task**	21.41 (2.81)	20.1 (3.58)	21.02 (3.23)	F_2,221_ = 2.35	0.07	0.94	0.23	0.03
**Eyes Task**	22.72 (4.81)	22.82 (4.39)	21.97 (5.56)	F_2,221_ = 0.44	0.96	0.81	0.66	0.04
**Advanced Theory of Mind**	12 (1)	12.03(0.57)	12 (1)	F_2,221_ = 0.14	0.98	0.65	0.75	0.005
***Emotional Sharing***								
**Affective Empathy (BES)**	37.29 (5.54)	36.93 (6.39)	37.48 (5.55)	F_2,221_ = 0.16	0.80	0.97	0.86	0.002
**Personal Distress (IRI)**	15.01 (5.05)	15.27 (5.77)	13.84 (5.04)	F_2,221_ = 1.44	0.96	0.42	0.28	0.001
**Empathic Concern (IRI)**	8.26 (3.81)	8.57(4.53)	8.1 (3.89)	F_2,221_ = 0.24	0.90	0.97	0.80	0.002

On the other hand, significant differences among groups during the post-reading phase in mentalizing measures were found, particularly in the first- and second-order false belief tests (F_2,211_ = 4.419; p = 0.001) and faces task (F_2,211_ = 4.068; p = 0.001). Specifically, post hoc comparisons of the interaction means indicated that the literary fiction group scored higher on the first- and second-order tests compared to the nonfiction group (p = 0.001) and science fiction group (p = 0.001), but no significant difference between nonfiction and science-fiction groups was found. In addition, the literary fiction group scored higher compared to the nonfiction group (p = 0.001) and the science-fiction group (p = 0.001) on the faces task. Empathic performance scores (means and SD) are reported in Table 3.

**Table 3:** Mean scores (and standard deviations) to the empathic performance separately for three groups. The results of the statistical analyses (ANOVA and post-hoc) are also shown. Statistically significant results are displayed in bold.

	Literary Fiction (G1)	Nonfiction (G2)	Science Fiction (G3)	Group1 vs Group2 vs Group 3				Effect Size
	*Mean (SD)*	*Mean (SD)*	*Mean (SD)*	*F*	p	p	p	*ŋ*^*2*^_*p*_
					G1 vs G2	G1 vs G3	G2 vs G3	
***Mentalizing***								
**Cognitive Empathy (EQ)**	13.6 (3.65)	13.04 (13.38)	14 (3.86)	F_2,211_ = 1.247	0.73	0.82	0.16	0.01
**Social Skills (EQ)**	6.85 (2.11)	6.38 (1.79)	6.25 (1.99)	F_2,211_ = 1.809	0.94	0.32	0.29	0.01
**False belief test**	7.06 (1.07)	5.74 (1.43)	5.94 (1.61)	F_2,211_ = 4.419	**0.001**	**0.001**	0.40	**0.16**
**Cognitive Empathy (MET)**	41.85 (8.21)	39.99 (6.97)	41.13 (4.4)	F_2,221_ = 1.416	0.31	0.57	0.29	0.012
**Faces Test**	17.39 (1.55)	15.71 (1.96)	16.04 (1.36)	F_2,221_ = 4.068	**0.001**	**0.001**	0.24	**0.17**
**Emotion Attribution Task**	43.78 (5.58)	42.80(6.27)	43.56 (7.03)	F_2,221_ = 0.486	0.61	0.95	0.56	0.004
***Emotional Sharing***								
**Emotional Empathy (EQ)**	12.92 (3.62)	12.42 (3.36)	13.09 (3.47)	F_2,221_ = 0.771	0.80	0.97	0.24	0.007
**Explicit Emotional Empathy (MET)**	5.47 (1.23)	5.17(1.33)	5.38 (1.23)	F_2,221_ = 1.021	0.34	0.85	0.36	0.009
**Implicit Emotional Empathy (MET)**	5.732(1.27)	5.27(1.3)	5.45 (1.23)	F_2,221_ = 0.392	0.90	0.94	0.35	0.004

## Discussion

Over the years, many attempts to improve the ability to empathize with other people both in healthy and psychiatric populations have been made. Kidd and Castano [17] opened a very interesting branch of research in the social cognition field linked to reading literary texts. Studies on emotional and social components involved during the reading of literary texts, however, are scarce, and only a few studies have specifically investigated the affective processes underlying story processing [12, 17].

On the basis of Kidd and Castano’s [17] and Black and Barnes’s research [16], we have tried to replicate and extend their studies in two important ways: (1) the participants underwent a large variety of empathy tests and (2) compared to previous studies [16, 17], our participants read a whole book and not only part of a book. Unlike reading a short story or an excerpt from a novel, reading an entire book may be more likely to promote narrative competence, which is defined as "the ability to recognize, absorb, interpret, and act on the stories and predicaments of others" [10, 11 (p. 3)].

In addition, in our study, participants completed a complex battery of social cognition tests before and after the reading phases. Our study, in line with previous studies [16, 17] showed that reading literary fiction enhances mentalizing abilities. The explanation of this result can depend on features of the literary fiction (i.e., this type of fiction allows readers to identify with the characters of the story without facing the potentially negative consequences of that involvement) [17]. Furthermore, characters are more often the primary focus of literary fiction texts, a feature that is likely to encourage readers to infer and track their mental states [17]. The science-fiction and nonfiction books did not have effects on any empathic competencies. Regarding science-fiction, we did not expect any effect because this genre narrates imaginative stories that are distant from reality and emotional contexts. We sustain that the pleasure in reading science fiction more often comes from imagining different realities than from understanding characters. Perhaps for this reason the science fiction genre is preferred by individuals with autism, and does not affect social skills [21]. On the contrary, regarding nonfiction books we expected effects on empathic abilities, particularly on emotional sharing competencies because this literary genre recounts real stories about dramas, pain or positive events that have marked the life of the character and that could involve the readers. The possible explanation of this result is that the nonfiction books could induce a "psychological block" in the readers because they know the events of the stories really happened [47]. Another possible explanation about the effect of literary fiction on mentalizing ability can derive from the main character of the book [16, 17]. In the literary fiction books selected for this study, the main characters have characteristics which are more similar to our readers compared to the characters of the other genres used in the present study. These aspects are important and essential in order to select adequate books when planning the treatment of a clinical population.

On the basis of the previous results [16, 17] and our results, we believe there is support for the notion that reading literary fiction may change how people think about the mental and emotional states of other people [18]. However, it does not appear to change our internal emotional sharing, that is the capacity to feel what people feel in a painful situation.

In general, our study showed the short-term effects of reading processes on mentalizing abilities. These results may set important goals for future low-cost rehabilitation treatment for several disorders in which a deficit of ToM is considered “core” to the disease such as in the treatment of individuals with schizophrenia [48] and autism [6].

However, our study has several limitations. Regarding the measures used, the mentalizing tests are more numerous compared to the emotional sharing measures, and the tests used during the pre- reading phase are different compared to the tests administered during the post- reading phase. Another crucial difference between mentalizing and emotional sharing measures is that the first are performance-based measures, while the second are self-report measures. In fact, according to Coman and Richardson' s study [49], these measures evaluate different aspects of cognitive and emotional functioning: self-reported functioning reflects the perception that a person has about his/her performance of tasks [49]; whereas, performance-based measures are more objective and assess the ability to perform a specific task correctly or incorrectly. We think that these differences depend on the “nature” of the construct that we evaluated through the tests. In our case, the mentalizing tests evaluate the subject’ s capacity to understand and recognize the mental and emotional states of other people. Thus the tests used evaluate the correct performance of the participants (for example, each subject can earn a score ranging from 0 -wrong response- to 1 -correct response- for each item); whereas, the emotional sharing tests evaluate the perception that the subject has about his/her own capacity to share the emotions of other people and right or wrong responses do not exist. For this reason, the best kind of emotional sharing test is a self-report one. In contrast to Djikic et al.’s [50] and Bal and Veltkamp’ s [47] results, in our study the fiction group showed significant differences compared to the other two groups in the performance-based measures. In our opinion, these results are more interesting because performance-based measures seem to estimate the real ability of the participants. On the contrary in the self-report measures, the results can to be overestimation or underestimation of actual ability. Based on these findings it can be said that reading literary fiction seems to improve mentalizing performance, but not the perception that the subject has of himself/herself compared to his/her capacity to understand the mental and emotional states of other people. In addition, the mentalizing tasks require a representation of the mental states of other people and they are based on the accurate decoding of specific cues, for example the facial expression and tone of voice. On the other hand, the emotional sharing tasks refer to internal or affective representation of another person’s emotional experience which can depend on real-world functioning, i.e., whether participants engage social skills in their daily life. Therefore, when a person predicts an emotional response in someone else he/she generates an internal affective representation of the predicted emotional response. The stronger the affective representation, the more likely he/she is able to experience empathy in the social context [51].

Another important limitation of the present study is the prevalence of female participants compared to male participants. This limit did not allow us to form conclusions about gender differences. In addition, the within-subject design can offer more detailed and accurate information, but it will be necessary to overcome the “learning effect” regarding the use of the same test during the pre- and post-reading phases. It will be interesting to evaluate the effect of literary fiction compared to other genres using different media, for instance movies. In addition, an important future perspective is to replicate our study in a group of adolescents with autism, using the same literary genres.

## Conclusion

In conclusion, the main objective of our research was to replicate Kidd and Castano’s [17] and Black and Barnes’s [16] studies, while overcoming their limits and highlighting the efficacy of narrative competences on social abilities. Recent research has shown that in psychological studies only 39% of original results are usually replicated [52]. Thus, we believe that our results confirm those of previous studies [16, 17] and strengthen the opinion that the efficacy of reading books on mentalizing abilities in healthy subjects.

We think that the present study has several benefits: the replication of the findings in a different culture/language, the use of different books, and the extension of the results using a greater number of measures. These characteristics are very important for the generalizability of the results.
